# HBV screening among West Africans living in the US: Influences of stigma, health literacy, and self-efficacy

**DOI:** 10.1097/HC9.0000000000000172

**Published:** 2023-06-02

**Authors:** Ponni V Perumalswami, Assita Belemkoabga, Lovely Joseph, Joel Erblich, Lina Jandorf

**Affiliations:** 1 Department of Medicine, Division of Gastroenterology and Hepatology, University of Michigan, Ann Arbor, Michigan, USA; 2 Institute for Healthcare Policy and Innovation, University of Michigan, Ann Arbor, Michigan, USA; 3 Department of Population Health Science and Policy, Icahn School of Medicine at Mount Sinai, New York, NY; 4 Department of Psychology, Hunter College and The Graduate Center, City University of New York, New York, USA; 5 Division of Gastroenterology, Department of Medicine, Icahn School of Medicine at Mount Sinai, New York, New York, USA

## Abstract

Applying theoretically informed constructs using an adaptation of the “Theory of Planned Behavior,” this study assessed social-cognitive and sociocultural determinants of HBV screening among West Africans living in the US to inform solutions to testing gaps.

**Methods:**

We developed and administered a theory-based survey in both English (41%) and French (59%) from September 2021 to April 2022 to a sample of West African-born individuals (n = 162). Predictors of HBV screening included: attitudes, perceived behavioral control or self-efficacy, and subjective norms along with health literacy (HL), language proficiency, and stigma of HBV infection. We hypothesized that these constructs would predict HBV testing. We also conducted path analytic modeling to better understand both direct and indirect effects of key factors on HBV screening status.

**Results:**

West Africans who completed the survey in English were younger with less education and lower income, whereas those who completed the survey in French reported higher HBV-related stigma. In a bivariate analysis of factors associated with HBV screening by language, less education was associated with lower HBV screening in English speakers. Adequate HL, higher self-efficacy, and higher English language proficiency were independently associated with HBV screening. Path analysis to better understand the interplay between social-cognitive and sociocultural factors revealed HL and stigma both had indirect effects on screening, mediated by differences in self-efficacy.

**Conclusions:**

This study identified HL and stigma as key indirect factors that influence HBV screening by way of self-efficacy in West Africans in the US. This work is a first step to identifying barriers that can lead to the development of an evidence-based intervention aimed at increasing HBV screening of West Africans to address health disparities.

## BACKGROUND

HBV infection is a leading cause of HCC, the third most common cause of cancer death worldwide.^1^ HBV is endemic in Africa where over 8% of the population 2,3 is estimated to be infected (vs <1% in the general population in the US^4^). Because most patients with HBV infections lack symptoms during the chronic phase, physicians have relied on screening for asymptomatic disease for early diagnosis. Fortunately, HBV can be detected with a simple blood test, and antiviral treatment effectively reduces disease progression.^5^ Despite the establishment of clear screening recommendations,^6^,^7^ ~75% of all HBV-infected persons in the US have not yet been diagnosed.^8^,^9^ Chronic HBV infection in the US is concentrated in immigrant populations (60%), of whom Africans (>10%) bear a great burden of this disease.^10^–^12^ African immigration has increased over the past 20 years.^13^ Over 2.1 million Africans reside in the US, with the highest numbers in New York, Texas, California, and Maryland.^13^

Health-seeking behavior for HBV testing is complex and not well understood, particularly in foreign-born communities living in the US. The “Theory of Planned Behavior” (TPB) is an evidence-based social-cognitive theory^14^ that was originally advanced to predict the intention to engage in a behavior.^15^ According to the TPB, the achievement of human behaviors such as HBV screening depends both on motivation and ability, which are influenced by a number of core considerations. The constructs in TPB include: (1) behavior beliefs (positive/negative attitudes) or the likely consequences of the behavior; (2) normative beliefs or the perceived social pressure or subjective norm; and (3) control beliefs or self-efficacy, the perceived ease or difficulty in performing the behavior. By linking one’s beliefs to behavior, TPB has been used to understand health behaviors such as sexually transmitted infection (STI) testing and cancer screening^16^,^17^ and develop evidence-based interventions to increase cancer screening and care among immigrants and minorities and reduce health disparities.^18^,^19^

In addition to the TPB elements listed, stigma, health literacy (HL), and language proficiency are considered key and related sociocultural determinants of engagement in health care, particularly in immigrant communities.^20^,^21^ Stigma plays an underrecognized role in influencing health behaviors.^22^ In studies of Asian and Asian-American communities, stigma has been found to be an important determinant for HBV screening and related care and treatment.^23^ Our prior qualitative work identified stigma as a potential factor that may influence HBV screening in West African communities.^24^ Inadequate HL has been shown to predict increased risk of long-term, life-limiting health conditions, and earlier mortality.^25^ People with lower HL are likely to rate their health as lower and to make more adverse lifestyle choices.^26^ Limited English proficiency (LEP) has grown in the last decade to 65 million persons, most of whom are immigrants.^27^ LEP has been associated with lower access to health care, fewer physician visits, lower use of preventive care, and a key factor associated with inadequate HL in immigrant communities.^28^–^30^ Addressing stigma, HL, and LEP are key to improving control over modifiable social determinants of health.

In this study, we aimed to identify theory-driven social-cognitive predictors of HBV screening in an understudied population, as well as key sociocultural factors (eg, HL, LEP, and stigma) relevant to immigrant populations. We, therefore, hypothesized that in West Africans living in the US: (1) HL, LEP, stigma, and knowledge of HBV would be important determinants of HBV screening, (2) based on the concept of linguistic relativity, also referred to as the Sapir-Whorf hypothesis,^31^ there may be important differences in sociocultural and social-cognitive factors based on language preference, and (3) that social-cognitive factors, especially self-efficacy, would underlie the effects of sociocultural factors on screening. Understanding the potentially complex interplay between social-cognitive and sociocultural factors in predicting HBV screening behavior has important practical implications, as it can guide the development of much-needed tailored and targeted interventions to promote HBV testing among West Africans.

## METHODS

### Participants and study population

We conducted a cross-sectional survey with West African-born community members residing in the New York City area. This study was conducted in accordance with both the Declarations of Helsinki and Istanbul and was approved by by the Icahn School of Medicine IRB under study IRB 18-00932 and University of Michigan HUM00191175. This study have a waiver of informed consent. We included participants who were 18 years or older, born in a West African country, and spoke either English or French. From September 2021 until April 2022, trained bilingual (French-English) study staff recruited and enrolled West African immigrants, to complete the survey. Study data were collected and managed using REDCap electronic data capture tools hosted at Icahn School of Medicine at Mount Sinai.^32^ REDCap is a secure, web-based software platform designed to support data capture for research studies, providing (1) an intuitive interface for validated data capture; (2) audit trails for tracking data manipulation and export procedures; (3) automated export procedures for seamless data downloads to common statistical packages; and (4) procedures for data integration and interoperability with external sources. Participants received a $20 gift card for their participation. This study was developed in collaboration with a community advisory panel of West African leaders (12 leaders representing the African diaspora communities), established in 2017, to help inform community outreach efforts. The panel reviewed the survey items for cultural appropriateness and helped inform recruitment of participants from community locations including: (1) our partner organization, African Service Committee (a non-governmental multiservice human rights agency dedicated to assisting immigrants, refugees, and asylees that provides walk-in services for health, housing, legal, social welfare, education, nutrition, and advocacy services to African immigrants), located in East Harlem New York,(2) places of worship, including mosques and churches in the New York City boroughs of Manhattan, Bronx, and Brooklyn, where West Africans are more densely located, and (3) community businesses, including taxi garages and hair salons. The recruited cohort for this study was a convenience sample rather than a consecutively recruited cohort as it relied on recruitment from the community sites where participants do not follow a specific workflow such as a clinic or health care setting. Our research coordinator who was bilingual English-French and African, made every effort to engage all potential subjects at our community recruitment sites and invited them to participate in this study in either language. All study procedures were approved by the Icahn School of Medicine Mount Sinai Institutional Review Board.

### Survey development and outcome measures

**FIGURE 1:**
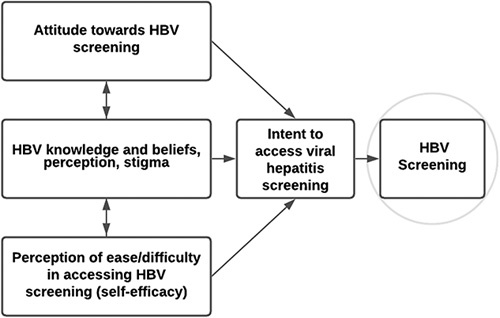
Theory or planned behavior framework adapted to HBV screening.

The primary objective of the study was to test the association of TPB constructs and key sociocultural factors in West Africans with HBV screening. Our primary outcome of interest was HBV screening. Our prior qualitative work in West Africans helped identify potential determinants of HBV screening that helped inform domains assessed in this study.^24^ The survey was developed using validated instruments or items used in prior studies to address the constructs in the overarching conceptual TPB framework focusing on individual attitudes and beliefs.^15^ Key TPB constructs included: (1) attitudes or views (positive/negative) toward HBV screening; (2) perceived subjective norm to assess knowledge, beliefs, perceptions, and stigma; and (3) perceived behavioral control to get screened, or the perceived ease or difficulty in performing the behavior (Figure 1). Perceived behavioral control, such as self-efficacy, is one’s confidence to successfully access screening and has been shown to predict HBV vaccination.^33^ The assessment also included previously validated sociodemographic questions (country of origin, time spent outside of the country of origin, and time spent in the US), language preference (when speaking with family and friends and with thinking 34) and separately language preference for health information (French, English, or other) and English proficiency;^34^ HBV awareness along with views on screening, current screening status, and intent to get screened; a validated measure of self-efficacy;^35^ an adapted measure of HBV knowledge from the Asian-American literature and used in our prior work;^36^ stigmas;^37^ and the short test of functional HL assessment (STOFHLA),^38^ a 7-minute assessment (and the only well-validated HL assessment available in French and English), allowing us to consider combining results from the 2 language groups. The entire assessment took <30 minutes to complete. All measures have strong reliability and validity (Supplemental Table S1, http://links.lww.com/HC9/A307). The survey was translated into French and back-translated, for measures not available in French. The West African community advisory panel reviewed this survey for cultural appropriateness and provided feedback that was incorporated to finalize the survey.

### Data analyses

Descriptive analysis including frequency (%) for categorical variables and mean with SD for continuous variables were used to summarize participant characteristics, HBV screening status, and the attitudes, beliefs, and perceived behavioral control to undergo HBV screening. We had a small number of surveys with some missing values (6.7%) and, therefore, column percentages were calculated based on the total number, which completed a response to an item. Student *t* tests and Chi-square (or Fisher exact as needed) were used for continuous and categorical variables respectively when comparing participant characteristics by language. Mantel-Haenszel Chi-square tests were used to test the association of factors stratified by language given the hypothesis of linguistic relativity on the primary outcome of HBV screening status. We set the 2-sided α to 0.05. All bivariate analyses were done using SPSS version 21.

To understand the direct versus indirect effects of sociocultural factors including HL and stigma on screening behavior, we also conducted a path analysis with self-efficacy as a mediator. Using MPlus Version 8.4, we estimated indirect pathways from both HL (STOHFLA) and stigma (“ashamed to have HBV”) to screening through self-efficacy, controlling for age and survey language. Although not anticipated, we also estimated possible correlations among HL, LEP, and stigma. The paths from HL and stigma to self-efficacy were modeled using a linear function, and the path from self-efficacy to screening behavior (binary outcome variable) was modeled using a logit-link function. Indirect effects were, therefore, parameterized in the log-odds scale. For ease of interpretation of linear parameters, we standardized the self-efficacy variable. We tested indirect effects for significance by estimating bootstrapped 95% CIs (*k* = 10,000 iterations). To estimate the model, we used maximum likelihood estimation with Monte Carlo integration.

## RESULTS

A cohort of 162 West African-born persons were recruited to complete the survey with 41.4% (n = 67) in English and 58.6% (n = 95) in French. Characteristics of the participants recruited are included in Table 1. The cohort was comprised of young adults with a median age of 35 (31, 44) years and 52.5% were women. In this cohort, most had completed more than a high school education (63.6%) and were used (61.1%), but earning <$25,000 per year. The median time spent in the US at the time of survey completion was 72 (36, 122) months. An assessment of general health-seeking behavior and awareness found 68.8% had <2 outpatient doctor visits per year and 22.7% indicated that they had postponed medical care in the last 1 year. In this cohort, 12.3% indicated that had health insurance and 76% had been previously tested for HBV with high rates of having heard of HBV. HBV-related stigma was reported by 30.1% who felt people should avoid people with HBV and 32.7% indicated they would feel ashamed if they had HBV. Inadequate HL was identified in 12.9%. Although English was chosen as the language of preference by most (60.6%) of the cohort, 84.8% and 72.8% indicated that non-English language was spoken with family and friends, respectively. While the majority of the survey results were complete, we had up to 18% missing data for specific variables (ie, self-efficacy) in the survey and, therefore, performed a sensitivity analysis between participants who completed the entire survey compared with those who did not. We did not find any factors to be significant difference among those who completed compared with those who did not.

**TABLE 1:** Baseline characteristics of the West African cohort (n = 162) by language preference.^a^

Characteristics	Overall cohort N (%)	English; n = 67 (41%)	French; n = 95 (59%)	*p*
Social demographics factors				
Age (median, IQR)	35 (31, 44)	34 (28,38)	37 (32,47)	0.006
Time in US in months (median, IQR)	72 (36, 121.5)	84 (48, 143)	68 (36, 106)	0.089
Sex (F)	85 (52.5)	35 (52.2)	50 (52.6)	0.989
Marital status				0.523
Married	72 (44.4)	32 (47.8)	40 (42.1)	—
Not married	90 (55.6)	35 (52.2)	55 (57.9)	—
Education level				0.003
High school or less	49 (32.2)	12 (18.8)	37 (42.1)	—
More than high school	103 (67.8)	52 (81.3)	51 (58.1)	—
Employment status				0.105
Employed	99 (61.1)	46 (68.7)	53 (55.8)	—
Unemployed	63 (38.9)	21 (31.3)	42 (44.2)	—
Income level				<0.001
<$25K	86 (55.5)	24 (36.9)	62 (68.9)	—
More than $25K	69 (44.5)	41 (63.1)	28 (31.1)	—
General health-seeking behavior and awareness				
No. doctor visits per year				0.864
3 times or more	50 (31.3)	20 (30.3)	30 (31.9)	—
Twice or less	110 (68.8)	46 (69.7)	64 (68.1)	—
Postpone medical visit				0.989
Yes	38 (23.8)	16 (24.2)	22 (23.4)	—
Hepatitis *B* awareness				
Heard of HBV; yes	153 (95.6)	62 (93.9)	91 (96.8)	0.448
Heard about HBV testing				0.803
Yes	142 (88.8)	58 (87.9)	84 (89.4)	—
Screened for HBV; yes	120 (74.1)	49 (73.1)	71 (74.7)	0.707
HBV stigma				
People should avoid people with HBV				0.289
No	109 (69.9)	48 (75.0)	61 (66.3)	—
Yes	47 (30.1)	16 (25.0)	31 (33.7)	—
I would feel ashamed if I had HBV				0.039
No	105 (67.3)	37 (57.8)	68 (73.9)	—
Yes	51 (32.7)	27 (42.2)	24 (26.1)	—
HL	20 (12.9)	6 (9.5)	14 (15.2)	0.339
Inadequate/marginal adequate	135 (87.7)	57 (90.5)	78 (84.8)	—
Self-efficacy median (IQR)	40 (36, 44)	40 (36, 47)	40 (35, 41)	0.059
English language proficiency				
Speaking level of proficiency				<0.001
Poor\fair	73 (45.6)	14 (21.2)	59 (62.8)	—
Good\excellent	87 (54.4)	52 (78.8)	35 (37.2)	—
Writing level of proficiency				<0.001
Poor\fair	69 (43.4)	16 (24.2)	53 (57.0)	—
Good\excellent	90 (56.6)	50 (75.8)	40 (43.0)	—
Reading level of proficiency				<0.001
Poor\fair	66 (41.3)	13 (19.7)	53 (56.4)	—
Good\excellent	94 (58.8)	53 (80.3)	41 (43.6)	—
Language preference for health information				<0.001
Not English	63 (39.4)	8 (12.1)	55 (58.5)	—
English	97 (60.6)	58 (87.9)	39 (41.5)	—
Language spoken with family				<0.001
Not English	134 (84.8)	47 (72.3)	87 (93.5)	—
English	24 (15.2)	18 (27.7)	6 (6.5)	—
Language spoken with friends				<0.001
Not English	115 (72.8)	33 (50)	82 (89.1)	—
English	43 (27.2)	33 (50)	10 (10.9)	—
Language used for thinking				<0.001
Not English	111 (71.2)	32 (48.5)3	79 (87.8)	—
English	45 (28.8)	4 (51.5)	11 (12.2)	—

*Note:* *p* value is a Fischer exact test rather than a chi-square.

a Column percentages were calculated based on completed responses (missing values excluded).

Abbreviations: HL, health literacy; IQR, interquartile range.

An analysis of participant characteristics stratified by surveys completed in English and French languages is shown in Table 1. Participants who completed the survey in English were significantly younger (*p* = 0.006), had more education (*p* = 0.003), and had higher income (*p* = 0.005). Most participants who completed the survey in English and French had previously completed HBV screening (73.1% and 74.7% respectively) and adequate HL (90.5% and 84.8% respectively) but English completers had significantly higher stigma and trended towards greater self-efficacy although not significant (*p* = 0.059).

To identify factors associated with HBV screening, we analyzed the entire cohort based on HBV screening status (Supplement Table S2, http://links.lww.com/HC9/A307) and then stratified by language (Table 2). In the univariate analysis stratified by language, we found that lower income was associated with participants who completed the survey in English and had not been previously tested for HBV (*p* = 0.034). Not surprisingly, persons who had heard of HBV testing were more likely to be screened for HBV (*p* = 0.003). Persons with adequate HL and higher self-efficacy were independently associated with screening for HBV in both language groups (*p* < 0.05).

**TABLE 2:** HBV screening status by preferred language.

	English		French		
HBV screening completed	No; n = 17	Yes; n = 49	No; n = 21	Yes; n = 71	*p*
Social demographics factors					
Age, mean(SD)	31.9 (8.1)	36.2 (9.5)	42.9 (15.1)	39.5 (10.3)	0.962
Time in the US, mean(SD)	111.7 (101.3)	104.1 (83.8)	71.3 (72.8)	88.2 (71.5)	0.675
Sex					0.322^a^
Male	10 (58.8)	22 (44.9)	10 (47.6)	35 (47.9)	—
Female	7 (41.2)	27 (55.1)	11 (52.4)	38 (52.1)	—
Marital status					0.202^a^
Married	6 (35.3)	25 (51.0)	8 (38.1)	31 942.5)	—
Not married	11 (64.7)	24 (49.0)	13 (61.9)	42 (57.5)	—
Education level					0.320^a^
High school or less	2 (11.8)	10 (21.3)	9 (42.9)	27 (40.9)	—
More than high school	15 (88.2)	37 (78.5)	12 (57.1)	39 (59.1)	—
Employment status					0.145^a^
Not employed	3 (17.60)	18 (36.7)	9 (42.9)	33 (45.2)	—
Employed	14 (82.4)	31 963.3)	12 (57.1)	40 (54.8)	—
Income level					0.034
<25K	10 (58.8)	14 (29.8)	13 (65)	49 (70)	—
More than 25K	7 (41.2)	33 (70.2)	7 (35)	21 (30)	—
General health-seeking behavior and awareness					
No. doctor visits					0.054^a^
Twice or less	15 (88.2)	31 (63.3)	13 (61.9)	51 (69.9)	—
3 times or more	2 (11.8)	18 (36.7)	8 (38.1)	22 (30.1)	—
Medical care postpone					0.937^a^
No	13 (76.5)	37 (75.5)	15 (71.4)	57 (78.1)	—
Yes	4 (23.5)	12 (24.5)	6 (28.6)	16 (21.9)	—
Hepatitis *B* awareness					
Heard of HBV					0.971^a^
No	1 (5.9)	3 (6.1)	1 (4.8)	2 (2.7)	—
Yes	16 (94.1)	46 (93.9)	20 (95.2)	71 (97.3)	—
Heard about HBV testing					0.003^a^
No	6 (35.3)	2 (4.1)	8 (38.1)	2 (12.1)	—
Yes	11 (64.7)	47 (95.9)	13 (61.9)	71 (97.3)	—
HBV stigma					
People should avoid people with HBV					0.064^a^
No	10 (62.50)	38 (79.2)	11 (52.4)	50 (70.4)	—
Yes	6 (37.5)	10 (20.8)	10 (47.6)	21 (29.6)	—
I would feel ashamed if I had HBV					0.188^a^
No	7 (43.8)	30 (62.5)	14 (66.7)	54 (76.1)	—
Yes	9 (56.3)	18 (37.5)	7 (33.3)	17 (23.9)	—
HL					0.015
Inadequate/marginal	0	6 (12.8)	5 (23.8)	9 (12.7)	—
Adequate	16 (100)	41 (87.2)	16 (76.2)	62 (87.3)	—
Self-efficacy, mean(SD)	36.7 (6.3)	41.3 (8.1)	33.7 (6.8)	39.8 (6.0)	<0.001
Hepatitis *B* knowledge, mean(SD)	58.4 (24.0)	63.5 (22.8)	52.0 (16.1)	59.1 (18.8)	0.962
English proficiency and language preference					
Speaking level of proficiency					0.484^a^
Poor/fair	3 (17.6)	11 (22.4)	14 (66.7)	45 (61.6)	—
Good/excellent	14 (82.4)	38 (77.6)	7 (33.3)	28 (38.4)	—
Writing level of proficiency					0.608^a^
Poor/fair	4 (23.5)	12 (24.5)	14 (66.7)	39 (54.2)	—
Good/excellent	13 (76.5)	37 (75.5)	7 (33.3)	33 (45.8)	—
Reading level of proficiency					0.557^a^
Poor/fair	3 (17.6)	10 (20.4)	13 (61.9)	40 (54.8)	—
Good/excellent	14 (82.4)	39 (79.6)	8 (38.1)	33 (45.2)	—
Language preference					0.336^a^
Not English	3 (17.6)	5 (10.2)	17 (81.0)	38 (52.1)	—
English	14 (82.4)	44 (89.8)	4 (19.00	35 (47.9)	—
Language spoken with family					0.130^a^
Not English	10 (58.8)	37 (77.1)	18 (90.0)	69 (94.5)	—
English	7 (41.2)	11 (22.9)	2 (10.0)	4 (5.5)	—
Language spoken with friends					0.287^a^
Not English	7 (41.2)	26 (53.1)	18 (90.0)	64 (88.9)	—
English	10 (58.8)	23 (46.9)	2 (10.0)	8 (11.1)	—
Language used for thinking					0.558^a^
Not English	8 (47.1)	24 (49.0)	16 (88.9)	63 (87.5)	—
English	9 (52.9)	25 (51.0)	2 (11.1)	9 (12.5)	—

a Column percentages were calculated based on completed responses (missing values excluded).

Abbreviation: HL, health literacy.

**FIGURE 2:**
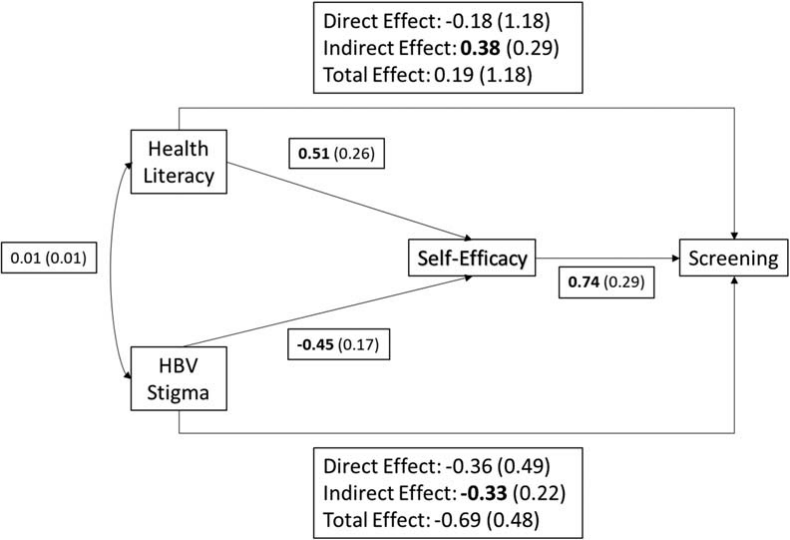
Path model of HL and stigma predicting screening behavior as mediated by self-efficacy. Parameter estimates and SEs are displayed. Estimates in bold are statistically significant. Abbreviation: HL, health literacy.

As outlined in the methods, we conducted a path analysis to estimate possible direct and indirect effects of TBP constructs and sociocultural factors on screening behavior. Consistent with the study hypotheses, we found a significant association between HL and self-efficacy (*b* = 0.51, SE = 0.26; *t* = 1.99, *p* < 0.05), such that individuals who exhibited adequate HL were predicted to have approximately one-half of an SD higher levels of self-efficacy than individuals with inadequate HL (Figure 1). Similarly, we found a significant association between stigma and self-efficacy (*b* = −0.45, SE = 0.17; *t* = 2.70, *p* < 0.007), such that individuals who reported stigma were predicted to have approximately one-half of an SD lower levels of self-efficacy than individuals who did not report stigma (Figure 2).

In addition, self-efficacy was significantly associated with screening behavior (*b* = 0.74, SE = 0.29; Wald *Z* = 2.50, *p* < 0.02), such that for every one SD increase in self-efficacy, the odds of having been screened more than doubled (OR = 2.09, 95% CI: 1.40, 4.40). Finally, we estimated the indirect effects of HL and stigma on screening through self-efficacy and found that both indirect pathways were significant. HL had a significant indirect effect on screening through increased self-efficacy (*b* = 0.38, SE = 0.29; bootstrapped 95% CI: 0.02, 1.12), such that, as accounted for by increased self-efficacy, individuals with adequate HL had 1.46 (bootstrapped 95% CI: 1.02, 3.06) times the odds of getting screened than those with inadequate HL. The direct effect of HL on screening behavior was not significant (*b* = −0.18, SE = 1.18; Wald *Z* = −0.16, *p* < 0.88).

Similarly, stigma had a significant indirect effect on screening through decreased self-efficacy (*b* = −0.33, SE = 0.22; bootstrapped 95% CI: −0.91, −0.06), such that, as accounted for by decreased self-efficacy, individuals who reported stigma had only 0.72 (bootstrapped 95% CI: 0.40, 0.94) times the odds of getting screened than those who did not report stigma. The direct effect of stigma on screening behavior was not significant (*b* = −0.36, SE = 0.49; Wald *Z* = −0.72, *p* < 0.47).

## DISCUSSION

With a growing focus on addressing liver cancer disparities, there is an important need to understand what influences critical upstream health-protective factors such as HBV screening. In this study, we aimed to evaluate the possible relationship of factors related to TPB along with other key sociocultural factors (HL and LEP) and HBV screening. The West African cohort was young and had more recently immigrated (<10 years in the US). This cohort had high HBV awareness and high rates of HBV screening (76% without significant differences by language) previously. Overall, nearly one-third reported HBV-related stigma with more stigma in English completers. Most participants had adequate HL (88%). Among the 59% of participants who completed the survey in French, most reported LEP. HBV awareness was higher in English completers. Participants with adequate HL, higher self-efficacy, and higher English language proficiency were each independently associated with screening for HBV in both language groups. In our path modeling, we found that self-efficacy is an important mediator of the effects of HL and stigma on HBV screening behavior. This study is the first to apply social-cognitive theory with key sociocultural factors to identify determinants of HBV screening as a health behavior in a growing population of English and French-speaking West Africans living in the US.

Self-efficacy is the belief of individuals in their ability to achieve a goal. Self-efficacy can affect health behavior in a number of ways, including determination of choice behavior; that is, which activities will be attempted, and which avoided.^39^ Self-efficacy also affects the amount of effort devoted to a task and the length of persistence when difficulties are encountered. For immigrant communities who may be variably connected to health care and encounter barriers in accessing care and treatment, self-efficacy, and factors that influence it can be significant and important for executing what we may consider straightforward health behaviors such as HBV testing. Self-efficacy has been found to influence testing for HBV in Asian-American communities^40^ and other infectious diseases including HIV among gay and bisexual men.^9^ Our study is the first to determine self-efficacy as an important factor that influences HBV testing in West African immigrant communities.

However, self-efficacy is complex and can be influenced by several factors that may indirectly influence HBV testing. One major finding in the study was the indirect effect of HL on self-efficacy with higher HL positively influencing HBV screening in West Africans. Certain populations are more likely to experience limited HL. These include racial and ethnic groups other than Whites, recent immigrants, and non-native speakers of English.^41^ This is because HL is influenced by language, socioeconomic status, cultural background, and past experiences such as with the health care system. There is limited data on the relationship between HL in patients with liver disease and engagement in liver care. A recent study has demonstrated the association of limited HL with frailty and reduced likelihood of listing for liver transplant in patients with advanced liver disease.^42^ A study among adults eligible for age-based screening in the UK examined the association between HL and colorectal cancer screening and found lower HL was associated with lower self-efficacy for colorectal cancer screening.^43^ In another study examining the relationship between HL and self-reported HIV medication, adherence researchers found that in a diverse cohort of patients across infectious disease practices in the US living with HIV, those with low HL had lower self-efficacy for taking their medications as prescribed.^44^ Our findings are consistent with these studies identifying HL as an important mediator of self-efficacy in care engagement. Of note, the definition of HL has been updated recently to not only include “personal HL” but also “organizational HL” or the degree, to which organizations equitably enable individuals to find, understand, and use information and services to inform health-related decisions and actions for themselves.^45^ As we begin to think about solutions to address HL needs in West Africans, it will be important to think about organizational and public health contexts, in which we seek to engage these communities and recognize that producers of health information and services have an important role in improving HL. Studies suggest that HL is a factor that can be modified with community-based interventions such as health promotion and enhanced navigation in foreign-born communities^46^ raising potential actionable targets for further studies to improve HBV screening in West African communities.

Another major finding in our study was the indirect negative effect of stigma on HBV screening by decreasing self-efficacy in West Africans. Stigma has been identified as a direct barrier to HBV screening in prior studies of Asian-American communities at-risk for HBV infection^47^ and as a potential barrier in our prior qualitative study in West Africans.^24^ Stigma more broadly has been identified as a factor that influences testing and care for infectious diseases, particularly STIs^37^ where health-related stigma may impact multiple domains such as social life, housing, and employment discrimination. Unfortunately, the stigma of STIs is common and has been shown to be in part promoted through public health campaigns in attempts to increase awareness in communities and reduce exposure.^48^ Furthermore, acquiring an STI can be associated with shame. Although HBV can be an STI, in many foreign-born communities living in the US it is transmitted vertically at birth from mother to child or in early childhood in countries where HBV has a much higher prevalence. Indirect paths for stigma on STI testing intention through self-efficacy have been reported among nonimmigrant groups.^17^ Stigma associated with HBV infection can be influenced by cultural context. In a study evaluating HBV stigma in Chinese immigrants, having a family member with HBV and higher HBV knowledge were associated with less stigma.^23^ Additional campaigns and health promotion to address stigma in foreign-born communities at-risk for and living with HBV have been effective at addressing and reducing stigma.^49^,^50^ Therefore, continued efforts focused on improving HL, addressing myths, and stigma will be important to enhancing efforts to increase HBV testing in at-risk West African communities.

This study had several strengths and limitations. We used a theory-informed approach to identify sociocultural factors associated with HBV screening in an understudied community with related liver cancer disparities. This work sought to understand determinants that influence HBV screening behavior to inform the development of evidence-based interventions to improve HBV testing in West African communities. In addition, we recruited participants that spoke the 2 major languages used by West African communities living in New York City, making our results more generalizable to West Africans. Before the study development, we had met with West African community leaders to solicit languages needed to reach their communities and leaders indicated that more than 90% of their community would be reached by studying populations who are either French or English-speaking. LEP was identified in most participants who completed the survey in French and was also found in a small number of patients when examining various constructs within the language proficiency assessment although this was not found to be a significant factor in the final analyses as it relates to HBV screening. As the survey was administered by study staff to participants (rather than self-administered), we included these results in our analyses. To our knowledge, this is the first reported assessment of HL and language proficiency in a growing West African immigrant population at-risk for chronic disease including HBV furthering our understanding of what factors may influence HBV screening behavior. Finally, we engaged with West African community leaders to develop and test study instruments and recruit participants from the community. A formal validation study of the newly developed survey translated into languages was not performed although where possible French language-validated instruments were incorporated such as short test of functional HL assessment, which is the only HL assessment validated in the French language. Developing ties to academic-community partnerships in communities that shoulder a disproportionate burden of HBV infection, particularly in immigrant communities where we have not historically built a lot of trust and partnerships, was an important implementation strategy that enabled us to conduct this study. Limitations of the study included a cohort that had mostly been screened for HBV although participants who had not been tested were also represented in 24% of the cohort. Identifying determinants of HBV screening behavior from both groups is important to have a complete understanding of barriers and facilitating factors. The characteristics of the cohort including a high proportion who had prior HBV screening were likely impacted by challenges in recruitment related to the ongoing COVID-19 pandemic. The recruitment period took longer than anticipated and we were not able to recruit participants from all the initially proposed avenues including community health fairs as many of these activities were paused with very slow reopening of community centers and places of worship to mitigate COVID-19 transmission risk. The recruited cohort for this study was a convenience sample as it relied on recruitment from the community over several months to achieve this sample size during COVID-19. In our experience, community-based recruitment for studies makes consecutive recruitment challenging. Our venues were local community sites where participants do not follow a specific workflow such as a clinic or health care setting. Our research coordinator who was bilingual English-French and African, made every best effort to engage all potential subjects at our community recruitment sites and invited them to participate in this study in either language. Finally, recruitment for this study (September 2021–April 2022) was challenging due to the impact of COVID-19 decreasing in-person interaction at community sites. By working with community leaders to identify the best places for the recruitment of participants, we were able to achieve this study sample.

Guided by theory, in this study, we have learned that HBV screening behavior among West Africans is related to self-efficacy, which is further indirectly mediated by important upstream factors of HL and stigma. Results from this study delineate the prevalence of sociocultural factors and test the association of these factors on positive views about HBV screening and intent to undergo screening. Understanding the interplay of beliefs and sociocultural factors that influence “simple” behaviors such as HBV screening in West Africans is the first step to developing evidence-based interventions such as effective health promotion that will improve HBV testing with the potential to reduce downstream liver cancer disparities. Culturally appropriate interventions are needed to reach West Africans with lower HL and address stigma related to HBV infection. Given the low rates of HBV testing among West Africans, interventions to address these sociocultural factors are critical to addressing significant HBV and related cancer disparities in West Africans. This study addresses an important research gap and has the potential to be used in the development of health promotion materials to reach a larger proportion of US-dwelling Africans. These factors will inform the development of a culturally targeted health promotion intervention that will be pilot tested as a next step.

## Supplementary Material
