# Supplementary material for: HBV screening among West Africans living in the US: Influences of stigma, health literacy, and self-efficacy
Source: Hepatol Commun. 2023 Jun 2;7(6):e0172. doi: 10.1097/HC9.0000000000000172 (PMC10241496; doi:10.1097/HC9.0000000000000172)
Supplement: Supplementary file 1 [file hc9-7-e0172-s001.docx]

Supplement Tables.

| **Table 1. Survey Measures** | | |
| --- | --- | --- |
| Measure | Source | Reliability |
| Demographics (standard items including SES, country of origin, insurance) | Jandorf et al., 2008; Jandorf et al., 2010 | Single item measure |
| English Proficiency and Language Preference | Gee et al., 2010 | r=0.87-0.92 |
| HBV awareness, HBV screening status, intent to get screened | Jandorf et al., 2008; Jandorf et al., 2010 | Single item measure |
| HBV Self-efficacy | Champion et al., 2005 | Alpha=0.87 |
| HBV Knowledge | Maxwell et al., 2010 | Alpha=0.94 |
| HBV Stigma | Maxwell et al., 2010 | 2-item dichotomous measure |
| HL STOFHLA | Connor et al., 2013 | Alpha=0.89 |

| **Supplement Table 2: Patient characteristics of entire cohort by HBV Screening status** | | | |
| --- | --- | --- | --- |
| **Characteristics** | **HBV Screening completed** | | **P value** |
|  | **No n (%) n=38** | **Yes n (%) n=122** |  |
| **I. Social Demographics factors** | | | |
| **Age mean(SD)n)** | 37.4 (9.8) | 38.4 (10.1) | 0.626 |
| **Gender** |  |  | 0.524 |
| Male | 20 (12.5) | 57 (35.6) |  |
| Female | 18 (11.2) | 65 (10.6) |  |
| **Marital Status** |  |  | .0326 |
| Married | 14 (8.7) | 43 (35) |  |
| Not Married | 24 (15) | 69 (56.2) |  |
| **Education Level** |  |  | 0.664 |
| High school or less | 11 (7.2) | 37 (24.5) |  |
| More than high school | 27 (17.8) | 76 (50.3) |  |
| **Employment Status** |  |  | 0.260 |
| Not employed | 12 (7.5) | 51 (31.8) |  |
| Employed | 26 (16.2) | 71 (44.3) |  |
| **Income level** |  |  | 0.375 |
| Less than 25K | 23 (14.9) | 63 (40.9) |  |
| More than 25K | 14 (8.9) | 54 (35) |  |
| **II. General Health Seeking Behavior and Awareness** | | | |
| **Number of doctor visits** |  |  | 0.452 |
| Twice or less | 28 (17.5) | 82 (51.2) |  |
| Three times or more | 10 (6.2) | 40 (25) |  |
| **Medical Care postpone** |  |  | 0.670 |
| No | 28 (17.5) | 94 (58.7) |  |
| Yes | 10 (6.2) | 28 (17.5) |  |
| **III. Hepatitis B Awareness** | | | |
| **Heard of hepatitis B virus** |  |  | 0.759 |
| No | 2 (1.2) | 5 (3.1) |  |
| Yes | 36 (22.5) | 117 (73.1) |  |
| **Heard about hepatitis B virus testing** |  |  | **<0.001** |
| No | 14 (8.7) | 4 (2.5) |  |
| Yes | 24 (15) | 118 (73.7) |  |
| **IV. HBV Stigma** | | | |
| **People should avoid people with hepatitis B (N=156)** |  |  | **0.047** |
| No Stigma (false) | 21 (13.4) | 88 (56.4) |  |
| Yes Stigma (True/ don't know) | 16 (10.2) | 31 (19.8) |  |
| **I would feel ashamed if I had hepatitis B (N=156)** |  |  | 0.117 |
| No Stigma (false) | 21 (13.4) | 84 (53.8) |  |
| Yes Stigma (True/ don't know) | 16 (10.2) | 35 (22.4) |  |
| **V. Health Literacy as measured by STOHFLA (n=155)** |  |  | 0.899 |
| Inadequate/marginal | 5 (3.2) | 15 (9.6) |  |
| Adequate | 32 (20.6) | 135 (87) |  |
| **VI. Self-efficacy (mean(SD)n)** | 34.7 (6.4) | 40.6 (7.1) | **<0.001** |
| **VII. Hepatitis B Knowledge (mean (SD))** | 54.2 (20.1) | 61.2 (17.1) | 0.054 |
| **VIII. English Proficiency and Language Preference** | | | |
| **Speaking Level of Proficiency** |  |  | 0.900 |
| Poor/Fair | 17 (10.6) | 56 (35) |  |
| Good/Excellent | 21 (13.1) | 66 (41.2) |  |
| **Writing Level of Proficiency** |  |  | 0.571 |
| Poor/Fair | 18 (11.3) | 51 (32) |  |
| Good/Excellent | 20 (12.5) | 70 (44) |  |
| **Reading Level of Proficiency** |  |  | 0.902 |
| Poor /Fair | 16 (10) | 50 (31.2) |  |
| Good/Excellent | 22 (13.7) | 72 (45) |  |
| **Language preference for health information** |  |  | 0.055 |
| Not English | 20 (12.5) | 43 (26.8) |  |
| English | 18 (11.2) | 79 (49.3) |  |
| **Language spoken with family** |  |  | 0.077 |
| Not English | 28 (17.7) | 106 (67) |  |
| English | 9 (5.7) | 15 (9.5) |  |
| **Language spoken with friends** |  |  | 0.415 |
| Not English | 12 (7.6) | 31 (19.6) |  |
| English | 25 (15.8) | 90 (56.9) |  |
| **Language used for thinking** |  |  | 0.702 |
| Not English | 11 (7) | 34 (21.7) |  |
| English | 24 (15.3) | 87 (55.7) |  |

*Percents were calculated based on completed responses (missing values excluded).
